# Optic Nerve Compression and Retinal Degeneration in *Tcirg1* Mutant Mice Lacking the Vacuolar-Type H^**+**^-ATPase *a*3 Subunit

**DOI:** 10.1371/journal.pone.0012086

**Published:** 2010-08-10

**Authors:** Nobuyuki Kawamura, Hiroyuki Tabata, Ge-Hong Sun-Wada, Yoh Wada

**Affiliations:** 1 Department of Biochemistry, Faculty of Pharmaceutical Sciences, Doshisha Women's College, Kyotanabe, Japan; 2 Division of Biological Sciences, Institute of Scientific and Industrial Research, Osaka University, Ibaraki, Japan; University of Florida, United States of America

## Abstract

**Background:**

Vacuolar-type proton transporting ATPase (V-ATPase) is involved in the proper development of visual function. Mutations in the *Tcirg1* (also known as *Atp6V0a3*) locus, which encodes the *a*3 subunit of V-ATPase, cause severe autosomal recessive osteopetrosis (ARO) in humans. ARO is often associated with impaired vision most likely because of nerve compression at the optic canal. We examined the ocular phenotype of mice deficient in *Tcirg1* function.

**Methodology/Principal Findings:**

X-ray microtomography showed narrowed foramina in the skull, suggesting that optic nerve compression occurred in the *a*3-deficient (*Tcirg1* ^−/−^) mice. The retina of the mutant mice had normal architecture, but the number of apoptotic cells was increased at 2–3 wks after birth. In the ocular system, the *a*3 subunit accumulated in the choriocapillary meshwork in uveal tissues. Two other subunit isoforms *a*1 and *a*2 accumulated in the retinal photoreceptor layer. We found that the *a*4 subunit, whose expression has previously been shown to be restricted to several transporting epithelia, was enriched in pigmented epithelial cells of the retina and ciliary bodies. The expression of *a*4 in the uveal tissue was below the level of detection in wild-type mice, but it was increased in the mutant choriocapillary meshwork, suggesting that compensation may have occurred among the *a* subunit isoforms in the mutant tissues.

**Conclusions:**

Our findings suggest that a similar etiology of visual impairment is involved in both humans and mice; thus, *a*3-deficient mice may provide a suitable model for clinical and diagnostic purposes in cases of ARO.

## Introduction

Vacuolar-type proton transporting ATPase (V-ATPase) is a multi-subunit complex formed from a membrane peripheral V_1_ sector and a membrane-spanning Vo sector. The V_1_ sector has catalytic sites for ATP hydrolysis, whereas the Vo sector is responsible for proton translocation [1]. Mammals express multiple subunit isoforms of the V-ATPase components in a tissue-specific manner [2]. The *a* subunit is a hydrophobic protein (approximately 100 kDa) and forms the Vo sector with proteolipids *c* and *c*“ subunits. The mammalian genome contains 4 genes for the *a* subunits, namely, *a*1–*a*4 [3], [4]. The *a*1, *a*2, and *a*3 subunits are present in various tissues at different levels. In contrast to the ubiquitous expression of the *a*1, *a*2, and *a*3 subunits, the expression of the *a*4 subunit is restricted to several types of ion-transporting epithelial cells [4], [5].

The *a*3 subunit of V-ATPase constitutes a transmembrane segment of the proton pump in late endosomes and lysosomes and functions in the luminal acidification of these organelles [3], [6]. Genetic defects in this subunit are responsible for a severe form of autosomal recessive osteopetrosis (ARO) in humans [7]. ARO is a life-threatening condition that causes increased bone density, which leads to decreased bone strength, resulting in multiple fractures and inflammation in bone tissues. V-ATPase with the *a*3 subunit is highly expressed in the cell surface of bone-resorbing osteoclasts and is responsible for acid secretion into the extracellular space between the osteoclasts and bone surface. Its deficiency causes ARO because of defective bone remodeling. This is also true for mice carrying mutations in the *Tcirg1* (also known as *Atp6V0a3*) locus, which encodes the *a*3 subunit [8], [9]. Reflecting the expression of the *a*3 subunit in various cells and tissues, *Atp6i* and *oc* mutations in *Tcirg1* cause a wide range of phenotypes, and the mice rarely survive for more than 1 month after birth [10]. The mutant animals exhibit malfunctions in systemic calcium homeostasis and develop rickets [11]. The *a*3 subunit is also required for the normal secretion of insulin and the bacteria-killing function of macrophages [12], [13].

Ocular complications are often associated with ARO in humans. This phenotype is thought to be a result of nerve compression at the optic canal because of the malfunction of bone resorption by osteoclasts [14]. The *oc*/*oc* mutant mice, which carry a mutation in the *Tcirg1*, are defective in the optomotor response, suggesting impairment of their vision. This defect can be corrected by bone marrow cell transplantation soon after birth, which supports the hypothesis that the dysfunction of osteoclasts in the hematopoietic cell lineage is responsible for the visual impairment [15]. While this study shows retrograde neurodegeneration is primary cause for the visual impairment in the *oc*/*oc* mutant mice, there remains other possibilities to be considered. The V-ATPase provides the ion motive force for aqueous humor formation [16] and is involved in the maintenance of acid–base regulation in epithelial cells of the ciliary body [17]. The degradation of the outer segments of the photoreceptor rods, an essential process for the regeneration of photoreactive opsin, requires V-ATPase driven phagosomal acidification of the retinal pigmented epithelium [18]. Recently, V-ATPase is shown to be involved in the proper development of the ocular system in flies and fish [19], [20]. These studies have shown that V-ATPase may be more directly involved in visual function. To understand the relevance of V-ATPase in the physiology and development of the ocular system, and to better understand the etiology of visual impairment associated with ARO, we examined the ocular defects in the *a*3 deficient mice. We also examined the expression pattern of each isoform of the *a* subunit in the murine ocular system.

## Results

### Retina archetecture in *Tcirg* ^−/−^ mice

**Figure 1:**
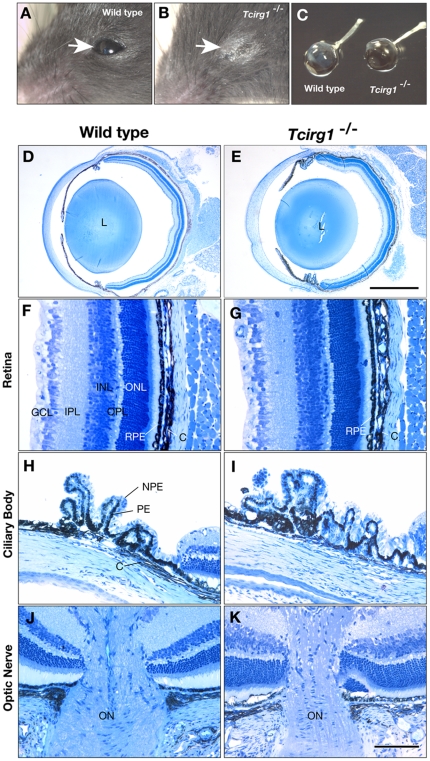
Ocular histology of wild-type and *Tcirg1* ^−/−^ mouse. Appearance of eyes of wild-type (A) and homozygous for *Tcirg1* ^−/−^ (B) at 3-wks after birth. The enucleated eye of a 3-wks *Tcirg1* ^−/−^ was practically same in size compared with that of the wild-type littermate (C). Technovit embedded eyes were sectioned and stained with toluidine-blue (D–K). Structure of retina (F, G), ciliary body (H, I), and optic nerve (J, K) were indistinguishable in the wild-type (F, H, J) and *Tcirg1* ^−/−^ (G, I, K) eyes. *L*, lens; *GCL*, ganglion cell layer; *IPL*, inner plexiform layer; *INL*, inner nuclear layer; *OPL*, outer plexiform layer; *ONL*, outer nuclear layer; *RPE*, retinal pigment epithelium; *C*, choroid, *NPE*, non pigmented epithelium; *PE* pigmented epithelium, *ON*, optic nerve. Bars, 1 mm (D and E) or 100 µm (F–K).

The *oc* mutation is a spontaneous mutation in the *Tcirg1* locus, which encodes the *a*3 subunit of V-ATPase [8]. Previous studies have shown that the *oc/oc* mice have near-normal electroretinograms [21]; however, they are defective in the optomotor response [15]. The *Tcirg1* ^−/−^ mutant mice carrying a deletion mutation frequently show eyes stained with stigma (Fig. 1A, B). This symptom appeared in animals of about 3 weeks of age, either in 1 or in both eyes. We examined the histology of the ocular tissues from wild-type and *Tcirg1* ^−/−^ mice and found no changes in the size of eye balls (Fig. 1C) and no obvious structural alterations in either the anterior or posterior parts of the ocular tissues (Fig. 1D–K), as was previously reported for *oc*/*oc* mutant mice [21]. The retinal layers were organized normally in the *Tcirg1* ^−/−^ mutant eyes (Fig. 1G). Retinal pigment epithelium (RPE) cells were present and there were no obvious structural changes in the uveal tissues rich in choroidal melanocytes. The ciliary bodies were also normal, with pigmentation in the pigmented epithelial cell layer of the *Tcirg1* ^−/−^ mutants (Fig. 1H, I), showing that melanin pigmentation occurs even in the absence of the V-ATPase *a*3 subunit [22]. The appearance of the optic nerve was similar in both genotypes (Fig. 1J, K).

**Figure 2:**
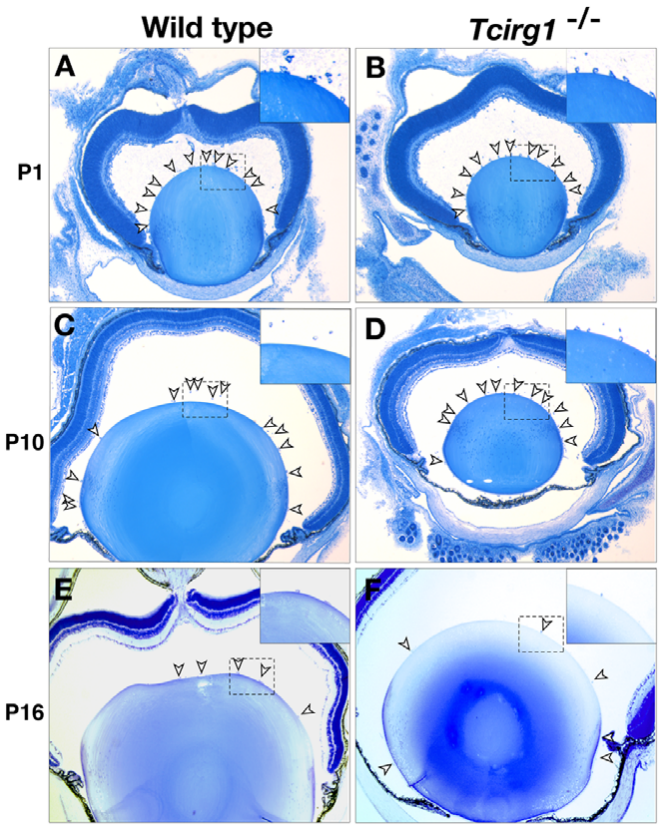
Regression of blood vessels in vitreous body in wild-type and *Tcirg1* ^−/−^ mouse. Wild-type (A, C, E) and *Tcirg1* ^−/−^ (B, D, F) ocular tissues were obtained at 1 (A, B), 10 (C, D), and 16 (E, F) days postnatal, and Technovit sections were stained with toluidine blue. Arrowheads indicate hyaloid vessels. The higher magnification images of hyaloid vessels in the boxed area are shown in the insets. No delay in hyaloid vessel regression was detected in *Tcirg1* ^−/−^ eyes.

Recently, we showed that the *a*3 subunit is required for the digestive function of macrophages [13]. Therefore, we considered the possibility that the loss of *a*3 might cause defects in vascular remodeling after birth, a developmental process that involves macrophage-like hyalocytes [23]. However, histological examination showed normal regression of the retinal and vitreous capillaries in the *Tcirg1* ^−/−^ mutant eyes (Fig. 2). Thus, the morphology of the eyes of both mutant and wild-type mice 3 weeks after birth was the same (Fig. 1D, E).

### Optic nerve compression in *Tcirg1* ^−/−^ mice

**Figure 3:**
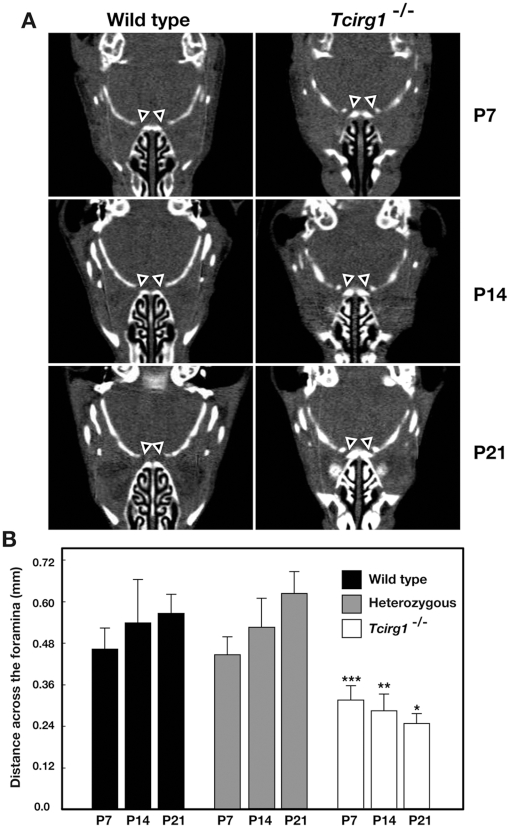
Narrowed optic foramen in *Tcirg1* ^−/−^ mouse. Micro-computed tomography scan sections of skull of wild-type and *Tcirg1* ^−/−^ mice at 1-wk (P7), 2-wks (P14) and 3-wks (P21) after birth (A). 3-D models were constructed and horizontal sections (caudal up, rostral down) generated by ImageJ software. Video S1 (wild-type) and S2 (*Tcirg1* ^−/−^) show the stack of the horizontal sections of 2-wks pups. (A) Images showing the widest opening at optic foramina indicated by white triangles. (B) Comparison of the distance across the optic foramina between wild-type and *Tcirg1* ^−/−^ or heterozygous mice. The distance across the foramina was measured by ImageJ software (wild-type, n = 10 (P7), n = 14 (P14) and n = 6 (P21); heterozygous, n = 18 (P7), n = 6 (P14) and n = 8 (P21); and *Tcirg1* ^−/−^, n = 6 (P7), n = 12 (P14) and n = 6 (P21)). Student's *t*-test (two-tails) was used for evaluating statistical significance between the wild-type and *Tcirg1* ^−/−^ litternates. *p = 0.00012, **p = 7.0×10^−6^, ***p = 1.86×10^−7^.

**Figure 4:**
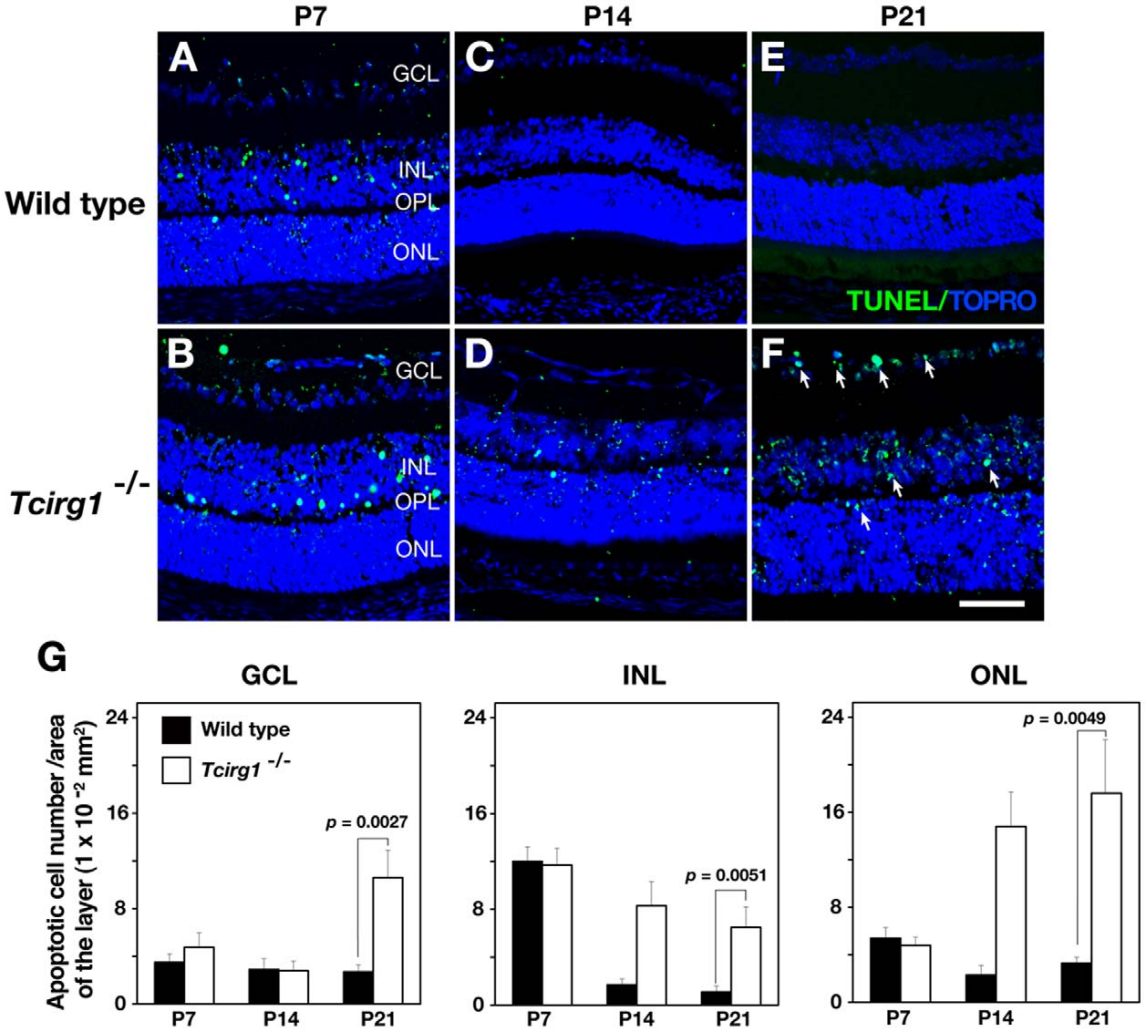
Degeneration of retinal layer in wild-type and *Tcirg1* ^−/−^ mouse. Apoptotic cells were detected by TUNEL assay (green) on cryosections of wild-type (A, C, E) and *Tcirg1* ^−/−^ mutant (B, D, F) tissues at 1-wk (P7), 2-wks (P14), and 3-wks (P21) after birth. The nuclei are shown with TOPRO-3 staining (blue). Three images of regions (1×10^5^ µm^2^) at retina were obtained from one section, and three sections were used for quantification in one eye. At least three eyes were examined in the experiments. Student's *t*-test (two-tails) was used for evaluating statistical significance. Bars, 50 µm.

The V-ATPase with *a*3 subunit is highly accumulated in the bone resorbing osteoclasts, and its deficiency causes severe osteopetrosis because of defective bone remodeling [24]. Impaired visual function in ARO patients is thought to be the result of nerve compression at the foramina [14]. We then examined the anatomy of the skulls of wild-type and *Tcirg1* ^−/−^ mutant mice by x-ray microtomography to determine whether this nerve compression also occurs in mice (Fig. 3 and Videos S1 and S2). In the mutant mice, the optic canals were narrower than in the wild-type or heterozygous mice throughout their postnatal development, suggesting impairment of foramina formation. The optic canals in the wild-type and heterozygous animals became wider after birth, however, those in the mutant animals were constricted, likely reflecting the defective bone resorption (Fig. 3B). The compression of the nerve often causes tissue degeneration. We examined the apoptosis in the retina during the postnatal development for 3-wks (Fig. 4). In wild-type animals, considerable cell death in retina took place one week after birth, then the cell death became occasional at later stages [25]. In the mutant retina, there remained numerous TUNEL-positive nuclei in the inner nuclear layer (INL) 3-wks after birth (Fig. 4). In the wild-type, the TUNEL-positive cells became less frequent in the ganglion cell layer (GCL) and in the outer nuclear layer (ONL) during the postnatal development, however, in the mutant retina, cell death increased in the ONL and GCL (Fig. 4G). GCL is the sites of nuclei of retinal ganglion cells whose axons leave the orbit through optic canal, the late onset cell death at this location suggested the presence of neuronal compression and axonal damage in the *Tcirg1* ^−/−^ mutant mice. Similar to our observation, it has been reported that optic nerve injury causes apoptosis in the retinal ganglion cells [26]. These results support the hypothesis that bony compression is causative for impaired vision in *Tcirg1* ^−/−^ mutant mice. In the mutant mice, the border between the INL and OPL (outer plexiform layer) appeared less smooth at 3-wks of age (Fig. 4E and F), whereas separation of the layers occurred normally during the postnatal development (Figs. 2 and 4).

### Distribution of the V-ATPase *a* subunits in ocular tissues

Our observation was compatible with the canonical view that bony compression at foramina causes neural degeneration in retrograde fashion, then brings loss of vision. However, it is also possible that V-ATPase is essential for the maintenance of retinal function directly. We examined the expression patterns of the *a*3 subunit in the ocular system, as well as the expression patterns of the other *a* subunits, because the presence of each V-ATPase *a* subunit in this tissue has not been well defined.

**Figure 5:**
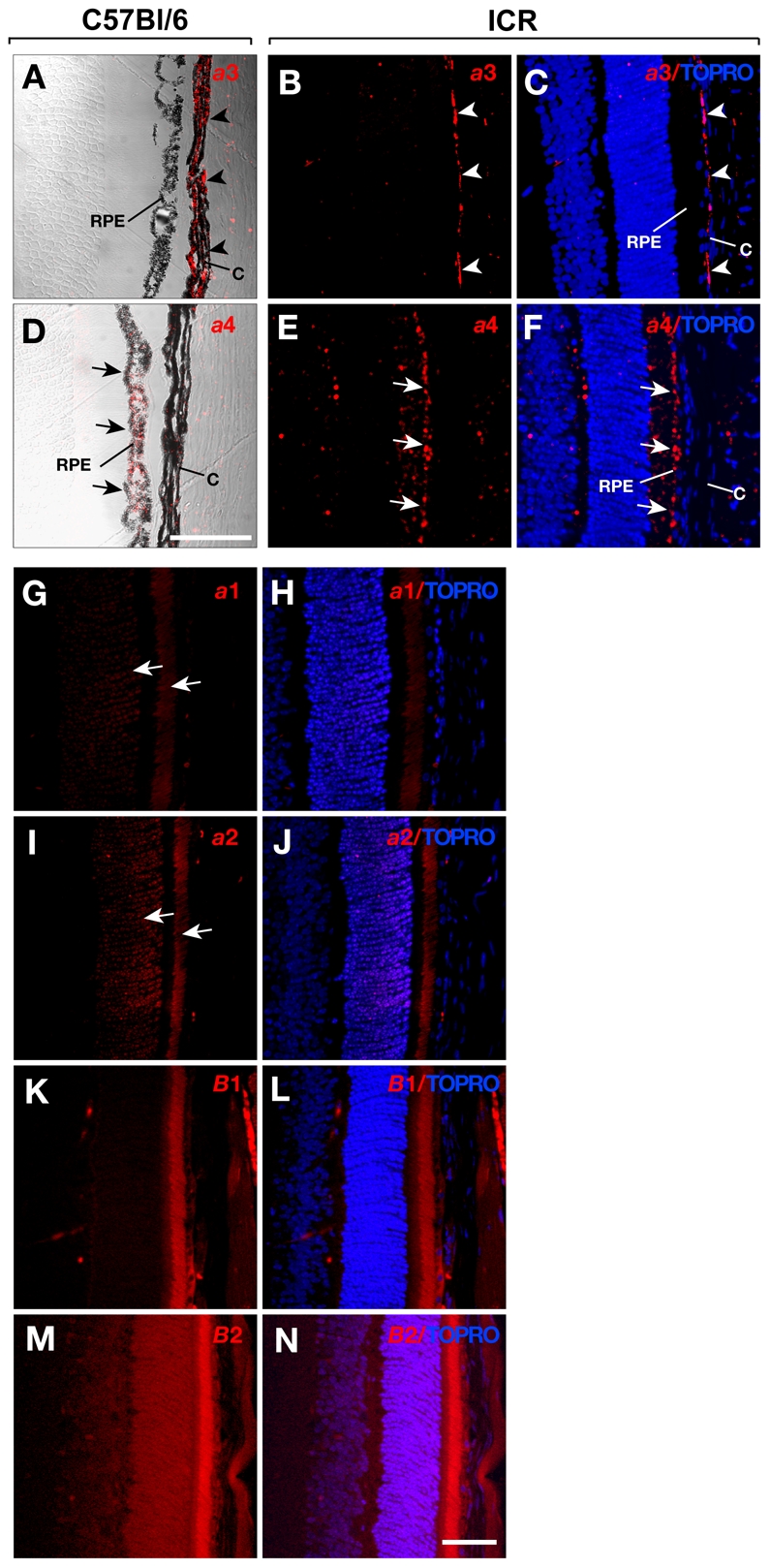
Expression of V-ATPase *a* subunits in retina. Technovit sections of C57Bl/6 black mouse (A, D, G–N) or ICR albino stock (B, C, E, F) were immuno-labeled for *a*1 (G, H), *a*2 (I, J), *a*3 (A–C), and *a*4 (D–F) subunits. The localization of *a*3 in choroid (*C*) is shown by arrowheads (A–C). The localization of *a*4 in retinal pigmented epithelium (*RPE*) is shown by arrows (D– F). The signals of *a*1 and *a*2 are also shown by arrows. Distribution of kidney-specific type *B*1 subunit (K, L) and ubiquitously expressed *B*2 subunit (M, N), constituting the V1 catalytic sector of V-ATPase were visualized as well. The nuclei are shown with TOPRO-3 staining (blue). Bar, 50 µm.

**Figure 6:**
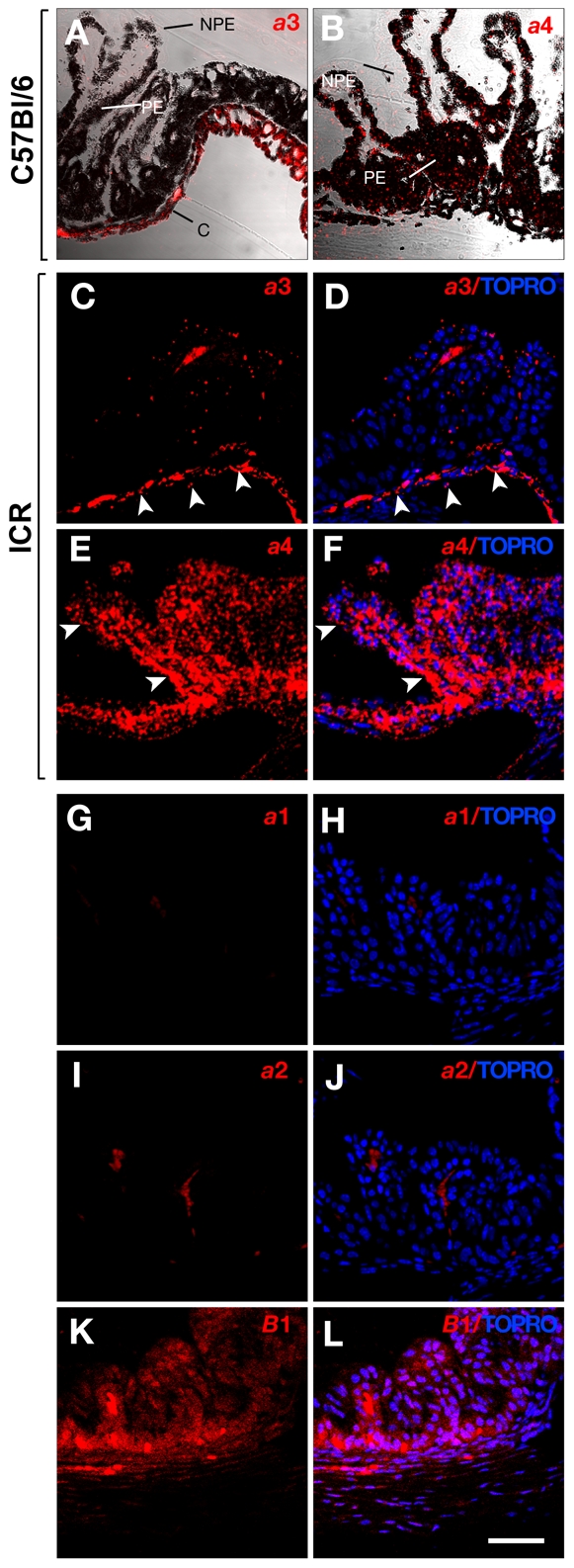
Expression of V-ATPase *a* subunits in ciliary body. Technovit sections of wild-type mice (C57Bl/6 black mouse, A, B and G-L; ICR albino stock, C–F) were immuno-labeled for *a*1 (G, H), *a*2 (I, J), *a*3 (A, C, D), *a*4 (B, E, F) and *B*1 (K. L) subunits. The nuclei are shown with TOPRO-3 staining (blue). *NPE*, non pigmented epithelium; *PE* pigmented epithelium. Bar, 50 µm.

We stained Technovit section of the eye with antibodies for each *a* subunit. The specificity and reactivity of the antibodies has been well established in previous studies [12], [22], [27], [28], [29]. In the photoreceptor layer of the retina, the *a*1 and *a*2 subunits were detected, but the expression of the *a*3 subunit was below the level that could be detected by immunofluorescence histology (Fig. 5). The *a*3 subunit was highly expressed in the choriocapillary meshwork between the RPE cells and sclera, but it was not found in the RPE cells (Fig. 5A–C). Weak but significant staining was also seen in ciliary bodies (Fig. 6 C–D). In the anterior part of the eye, the *a*3 subunit was found in the capillary-rich tissue between the muscle and nonpigmented epithelial (NPE) and pigmented epithelial (PE) layers (Fig. 6A, C, D).

The ocular ciliary epithelium is known to express the *B*1 subunit of V-ATPase on the cell surface [16]. The *B*1 subunit is expressed specifically in epithelial cells. The presence of *B*1 suggests that the V-ATPase in ciliary epithelium is composed of a combination of specific subunit isoforms, because V-ATPases resides on the plasma membrane of renal epithelial cells assemble preferentially with the *a*4 and *B*1 subunits in the Vo and V1 sectors, respectively [30]. Indeed, we found that the *a*4 subunit was present in the PE cells (Fig. 6B, E) and that this expression pattern was similar to that of the *B*1 subunit (Fig. 6K, L). In contrast, the signals for the *a*1 and *a*2 subunits were faint and barely detectable above background levels (Fig. 6G–J) in the ciliary bodies. The accumulation of melanin in cells such as NPE cells can interfere with the immunofluorescence detection of V-ATPase isoforms. We compared the eyes of C57Bl/6 (black) and ICR (albino) mice. As shown in Figs. 5 and 6, antibodies against the *a*3 and *a*4 subunits gave similar staining patterns in the retina and ciliary bodies from both the black and albino mice; therefore, the lack of immunofluorescence signal was not because of masking by the black pigment.

The ciliary NPE is considered to be an anterior extension of the photoreceptor layer, whereas the ciliary PE represents a continuation of the RPE of the retina. Consistent with this tissue architecture, the *a*4 subunit, which was shown to be expressed in the PE of the ciliary epithelium, was highly expressed in RPE cells (Fig. 5D–F). This distribution is similar to that of the *B*1 subunit (Fig. 5K, L).

**Figure 7:**
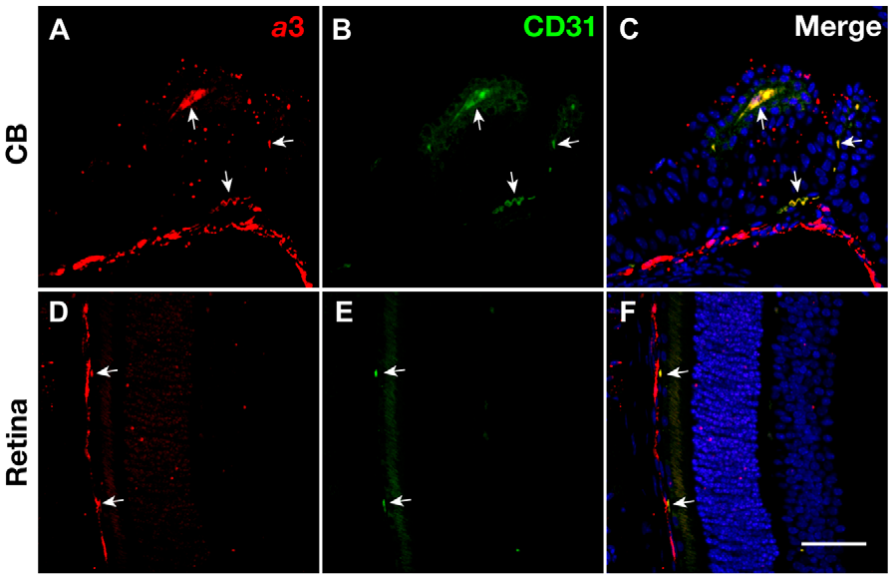
Presence of V-ATPase *a*3 subunit in endothelial cells. Technovit sections of wild-type mouse were double-immunostained with anti-*a*3 (red, A and D) and anti-CD31 (green, B and E) antibodies. Merged fluorescent signals are shown in C and F, where the nuclei are shown with TOPRO-3 staining (blue). Bar, 50 µm.

**Figure 8:**
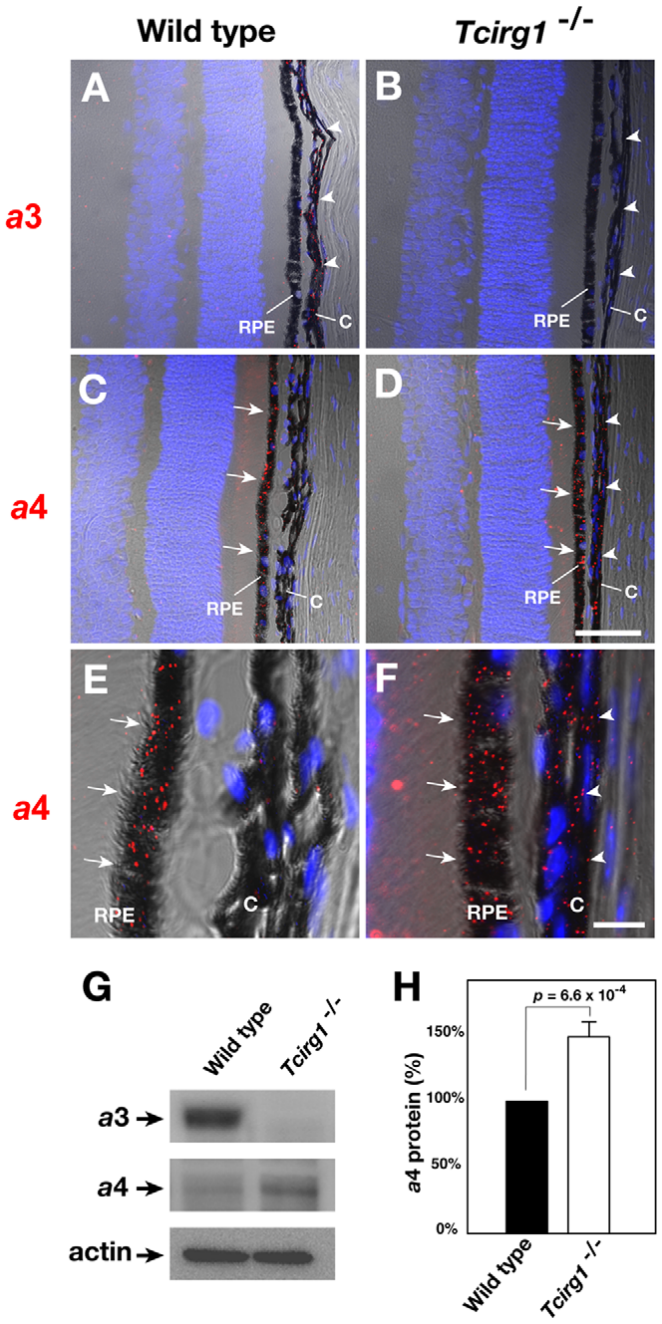
Ectopic expression of *a*4 subunit in uveal choriocapilary meshwork of *Tcirg1* ^−/−^ mouse. Technovit sections of wild-type (A, C, E) and *Tcirg1* ^−/−^ eye were stained with anti-*a*3 antibodies (A, B) or anti-*a*4 antibodies (C–F), and then viewed under laser microscope. TOPRO-3 (blue) were used for counter stain of nuclei. *RPE,* retinal pigmented epithelium; *C,* choroid. Bars, 50 µm (A–D) or 10 µm (E and F). Comparison of protein levels of *a*3 and *a*4 in RPE/choroids of wild-type and of *Tcirg1* ^−/−^ mice by immunoblotting (G). The amounts of *a*4 subunits on the immunoblots were quantified by a lumino-image analyzer (n = 3)(H). Expression of *a*4 is up-regulated in *Tcirg1* ^−/−^ RPE/choroids.

In the ciliary body, CD31, a marker protein for the capillary endothelium, was colocalized with the *a*3 subunit. A part of the *a*3 signal overlapped with CD31 in the choriocapillary meshwork underlying the retina (Fig. 7D–F). These results indicated that the *a*3 subunit was highly expressed in the capillary tissues. In addition to the endothelial cells, stronger signals were observed in the CD31-negative cells in the choroid (Fig. 7A, D). The signal in the choroid disappeared upon the loss of the *a*3 subunit in *Tcirg1* ^−/−^ mice (Fig. 8B). Interestingly, in the *Tcirg1* ^−/−^ mutant tissues, the *a*4 subunit of V-ATPase was present in the choriocapillary meshwork, while the expression of the *a*4 subunit was virtually absent in the wild-type cells (Fig. 8C–F). Immunoblotting analyses on RPE/choroid layer confirmed that the amount of *a*4 subunit increased approximately 1.5-folds in the *Tcirg1* ^−/−^ mice (Fig. 8G). These results suggested that the function of *a*3 in choroid could be compensated by the *a*4 isoform.

## Discussion

V-ATPase with the *a*3 isoform functions as a proton-secreting system in the plasma membrane of osteoclasts [6]. The loss of this protein results in a characteristic phenotype of osteopetrosis in both humans and mice, demonstrating its essential role in extracellular acidification and bone resorption. Osteopetrosis is often associated with impaired vision. Defective formation of the foramina, where the optic nerve passes, is presumed to be the cause of the impaired vision. In this study, we showed that mice lacking the *a*3 subunit have a narrowed optic canal. Although the retina of the mutant mice had normal architecture, the number of apoptotic cells was increased. These observations are consistent with the view that retrograde retinal degeneration occurs due to optic nerve compression.

We showed that the *a*3 subunit is expressed in extraretinal tissues, i.e., uveal choriocapillary meshwork. The capillary system in these tissues is known to be fenestrated; thus, small molecules and solutes can pass through without cellular activity but large molecules must be transported actively by transcytosis involving the endocytic and recycling pathways [31]. V-ATPase with the *a*3 subunit is a component of endosomes and lysosomes; thus, its activity may be important in these exchange processes. The expression pattern of the *a*3 subunit raises a possible scenario that V-ATPase with *a*3 may play a role in exchanging material across the endothelial cells, and loss of this function may result in increased cell death in the INL and ONL, where the nuclei of interneurons and photoreceptors reside, respectively, and may affect the OPL, the layer containing synaptic contacts between the interneuron and photoreceptor cells. Although the expression levels of *a*3 subunit are low in the retina, minor amounts of V-ATPase with *a*3 subunit is likely to be associated to the endosomes and lysosomes, thus it is also possible that dysfunction of these endocytic compartments may lead increased cell death in retinal tissue.

One form of osteopetrosis results from the loss of the anion-conducting channel, Clc-7. The loss of the *clcn-7* gene is accompanied by severe impairment of visual function and atrophy in the neural retina, due to the loss of lysosome function in the neural retina and RPE [21], [32]. Mutations in the *Tcirg1* gene, which encodes the V-ATPase *a*3 subunit, account for 50% of human ARO cases. Combined with mutations in *Clcn7* (chloride channel), genetic defects in these genes are responsible for two-thirds of cases of ARO [33]. Although defective vision is associated with both mutations, the results of our study and those of previous studies suggest that the underlying mechanisms are different, because V-ATPase with the *a*3 subunit is less abundant in RPE cells or in other part of retina, whereas the Clc-7 protein is highly expressed in neural tissues [21]. The loss of the V-ATPase *a*3 subunit results in early mortality (∼3 weeks after birth) compared to the *clcn7* mutants that survive for 2–3 months; therefore, the *a*3 mutant mice may not survive long enough to develop retinal degeneration. Hematopoietic stem cell transplantation (HSCT) is one of the first choices for the treatment of ARO and lessens the severity of V-ATPase *a*3 mutation both in humans and mice [15], [33]. However, HSCT may not be an effective treatment for ARO patients with Clc-7 dysfunction, who often also have neurological problems due to defective lysosomal function in neuronal cells [33], [34]. This implies that precise genetic diagnosis is necessary to determine the benefit of HSCT in patients with ARO.

In this study, we found that the *a*4 subunit was highly expressed in PE and RPE cells. Despite the relatively high expression of the *a*4 subunit in retinal tissues, there have been no reported cases of visual impairments due to the loss of *a*4 function in humans. This may be because the other *a* subunit(s) compensate the lack of *a*4. There have been no reports of mutant mice lacking *a*4 function. Similar to the loss of *a*4 function, mutations in the *ATP6V0A2* locus, which encodes the *a*2 subunit, do not seem to cause visual impairment, whereas the loss of the *a*2 subunit results in an abnormal assembly of the extracellular matrix and skin (cutis laxa), probably because of defective posttranslational glycosylation [35]. Because the retina expresses several *a* subunits in combination, functional subunits may compensate for the loss of a single subunit; thus, loss of either *a*2 or *a*4 alone may not be sufficient for the development of a defective visual phenotype. We have shown the upregulation of the *a*4 subunit in the retinal layer of *a*3 knockout mice, while the *a*4 subunit was below the detectable level in the wild-type animals. Along with this current finding, the *Tcirg1* ^−/−^ mice accumulates the *a*2 subunit in the islets of Langerhans [12]. These results suggest the presence of mechanism compensating a loss of particular subunit isoform with the other isoforms, adding further layers of complication in genotype/phenotype relationships. This hypothesis will be addressed by creating compound mutants with mutations in each of the *a* subunits.

## Materials and Methods

### Antibodies, reagents, and animals

Specific antibodies against each isoform of the mouse V-ATPase subunit *a* have been described previously [27], [30]. Anti-*B*1 and *B*2 antibodies have been described previously [30]. Fluorescent dye–conjugated secondary antibodies were obtained from Jackson ImmunoResearch. C57Bl/6 and ICR mice were purchased from SLC Japan. The modification of the *Tcirg1* locus and creation of mutant mice (*Tcirg1* ^−/−^) have been described previously [13]. All experiments involving animals were conducted in accordance with the institutional guidelines of the Institutional Animal Care and Use Committees of the Institute of Scientific and Industrial Research (ISIR), Osaka University and the Committee of Doshisha Women's College (DWC). The animal experiments were approved by the Committees (Dosan19-01-0 at ISIR, Osaka Univ. and Y09-018 at DWC).

### Immunohistochemistry and immunoblot analysis

Tissues were dissected and fixed overnight at 4°C with 4% formaldehyde in 0.1 M sodium phosphate buffer (pH 7.2), which was freshly made from paraformaldehyde (Sigma-Aldrich). The fixed tissues were embedded in Technovit 7100 resin after dehydration through an ethanol series and cut (3 µm thickness) by means of a rotary microtome. The sections were incubated for 1 h at 4°C in a PBS-based blocking buffer containing 0.2% gelatin, 0.2% saponin, 1% bovine serum albumin, and 1% normal goat serum. Subsequently, the sections were incubated overnight at 4°C with antibodies diluted in the blocking buffer. After being washed with the blocking buffer, the sections were incubated with the appropriate secondary antibody for 1 h at room temperature and extensively washed with PBS.

For in situ TUNEL assays, the fixed tissues were embedded in OCT compound (Sakura Finetech Japan), and retinal cryosections (5 µm thickness) were obtained by using a cryomicrotome. The sections were processed for TUNEL with fluorescein-dUTP (Roche) and counterstained with TOPRO-3 (Invitrogen). The slides were mounted in VectorShield mounting medium and examined under a confocal microscope (Zeiss LSM-510).

The RPE/choroids was isolated under stereomicroscope and lysed in extraction buffer containing 50 mM Tris-HCl (pH 7.4), 1% SDS plus Complete proteinase inhibitors (Roche) and 1 mM phenylmethylsulfonyl fluoride by sonication for 20 sec. The protein concentration of the lysate was determined by the BCA colorimetric assay (Pierce). The lysates (20 µg protein) were run through 5–20% SDS-polyacrylamide gels, transferred onto PVDF membrane and probed with the primary antibodies and horseradish peroxidase conjugated secondary antibodies (Jackson Immunoresearch). The blots were developed by ECL system (GE Healthcare) and images were obtained and quantified in LAS-1000 lumino-image analyzer (Fuji Film). The β-actin was detected with a mouse anti-β-actin antibody (Abcam) and used as the internal marker.

### Computed tomography and modeling

Mice were anesthetized with ketamine and xylazine, and their bone anatomy was scanned using a microCT scanner (Latheta LCT-100, Aloka) with 0.06 mm ×0.06 mm ×0.06 mm resolution. The 3-D models were generated by NIH ImageJ software on a Macintosh computer. The maximum opening of foramina was measured on coronal sections on the ImageJ software by a person who did not share genotype information of the specimen. Statistic significance was evaluated with Student's *t* test on Microsoft Excel program.

## Supporting Information

Video S1 Foramina of wild-type mouse.
(0.38 MB AVI)

Video S2 Foramina of mutant mouse.
(0.35 MB AVI)
